# Qualitative changes in children’s physical activity and sedentary behaviours throughout the COVID-19 pandemic: The HomeSPACE project

**DOI:** 10.1371/journal.pone.0280653

**Published:** 2023-01-20

**Authors:** Amie B. Richards, Michael P. Sheldrick, Nils Swindell, Harriet G. Barker, Joanne Hudson, Gareth Stratton

**Affiliations:** Applied Sports Technology Exercise and Medicine (A-STEM) Research Centre, Swansea University, Swansea, Wales, United Kingdom; Kent State University, UNITED STATES

## Abstract

Opportunities for children to be physically active during the COVID-19 pandemic were limited, resulting in a decrease in overall physical activity and an increase in sedentary behaviour during the lockdown restrictions of the pandemic. This study further explored these changes across various stages of the restrictions, starting during the first UK-wide lockdown in March 2020 through to the “new normal” in December 2021. Nine families, consisting of eleven children (36% girls, 64% boys; aged 13.38 years ± 1.14), eight mothers and one father were tracked throughout this time, using semi-structured interviews to explore the fluctuations in physical activity and sedentary behaviour in the home environment in the context of self-determination theory. Findings indicate that as restrictions eased, physical activity within the home decreased, as children were exposed to more opportunities at school and in the community; these opportunities seemingly increased children’s motivation to be physically active through increasing levels of their basic psychological needs of autonomy, competence, and relatedness. Some children’s physical activity levels have returned to pre-COVID-19 levels, with a newfound enjoyment for being physically active. Whilst others now prefer to pursue more sedentary behaviours that became habitual during the lockdown restrictions. Accessible opportunities now need to be promoted to drive up children’s motivations to be physically active following the years of uncertainty around the COVID-19 pandemic.

## Introduction

The COVID-19 pandemic provided a natural intervention [1] on children’s physical activity (PA) and sedentary behaviour (SB). COVID-19 restrictions constrained children’s movement resulting in them spending a considerable amount of time in the home environment. Health-related research has demonstrated the importance of PA in reducing the risk of severe COVID-19 disease [2]; despite this, children’s PA levels within the United Kingdom remain at low levels [3]. Therefore, it is important to understand how factors during the varying levels of restrictions influenced PA and SB and how these factors may impact on physical and sedentary behaviours in the future.

PA has been well established as a powerful contributor to improving children’s physical and mental health [4] and plays a significant role in addressing specific physical and mental health issues including obesity [5] and depression [6]. Despite this, only 17.5% of children in the UK meet the national recommendation of 60 minutes of moderate to vigorous PA per day [3]. Subsequent reports found PA decreased further during the COVID-19 pandemic. These reports found sports activity declined and in contrast, recreational screen time increased [7–9], with an overall decline in children’s PA [10]. Of even greater concern is short-term decreases in PA levels and increases in SB may become permanent [11], supported by evidence that objectively measured PA levels decreased, and SB remained high in children, even when schools were reopened [12]. Due to the COVID-19 restrictions, children spent nearly all their time within the home environment. Our previous research with the HomeSPACE project has clearly identified the barriers and facilitators in children’s home environments that influence children’s PA and SB [7] but has not explored how changing environments impacted these.

The Self Determination Theory (SDT) can be used as an interpretive framework to understand factors that predict PA or SB [13] with extensive application in this context [14] and more recently, with health-related behaviours during the COVID-19 pandemic [15, 16]. SDT is a control-based theory [17] comprising of six mini-theories. The Basic Psychological Needs Theory (BPNT) is one of these and argues that optimal functioning is predicted by autonomy, relatedness, and competence. These three psychological needs assist in the understanding of the motivation and engagement of individuals. SDT identifies that social and contextual factors play a part in determining an individual’s level of motivation to be active. COVID-19 restrictions on activity became a key contextual factor influencing motivation during lockdown. To further develop the theoretical background, differences between an autonomy supportive environment and a controlling environment have been highlighted [18]; and COVID-19 restrictions posed a controlling environment by restricting choice for individuals’ opportunities to be physically active.

Our initial qualitative HomeSPACE study explored children’s PA and SB during the first national lockdown of the COVID-19 pandemic and identified several barriers and facilitators to PA within multiple levels of the socioecological model [7]. The identified barriers and facilitators included gender, siblings, home-schooling, access to equipment/facilities and COVID-19 laws and regulations. This initial study reported compelling evidence that COVID-19 was significantly impacting on children’s health promoting activity, warranting further longitudinal exploration. Moreover, insufficient attention has been paid to changes in children’s PA and SB across various stages of the lockdown restrictions in the UK. The aim of this study was therefore to track changes in experiences of children’s PA and SB across time related changes in COVID-19 pandemic restrictions in the context of the SDT.

## Methods

### Study design & participants

The HomeSPACE project was a cross-sectional longitudinal observational study exploring the influence of the home environment on children’s PA levels and SB [19]. The HomeSPACE COVID-19 project is a longitudinal study investigating changes within home environments through the COVID-19 pandemic and the impact of this on children’s PA and SB. During the second, third and fourth phases of the HomeSPACE COVID-19 project, semi-structured, online interviews took place with families to investigate changes in PA and SB within the home because of the fluctuating COVID-19 lockdown restrictions between March 2020 and December 2021.

Recruitment methods have been previously described [7], including the incentive of receiving a £20 voucher to participate in the research. Briefly, the 103 families who participated in the wider HomeSPACE COVID-19 project were categorised into low (1–636), medium (637–1273) and high (1273–1909) tertiles from their score in the Welsh Index of Multiple Deprivation (WIMD) as previously described [7]. The quantitative element of the HomeSPACE project measured sitting time, house size, moderate to vigorous PA (MVPA) and garden size; these were stratified within each SES into low, medium, and high. The programme R^©^ was used for random sampling to select an equal number of participants from each stratum using the “stratified” function. These selected participants were then contacted via telephone and email. Twenty families agreed to participate in the first interviews; there was some attrition into the second phase of interviews where 14 of the original families took part and the third round of interviews where nine of the original families took part. Attrition occurred due to natural drop-out and families being unwilling to remain involved for the duration of the project. For the purposes of this study, only the nine families who took part in all three phases of interviews are used for analysis. The nine families consisted of 11 children (36% girls, 64% boys; aged 13.38 years ± 1.14), one father and eight mothers. There was an uneven split between SES groups due to the final sample of volunteer participants, with six families from high SES backgrounds, one from a medium SES and two from low SES backgrounds.

### Data collection

The study received approval from the institutional Research Ethics Committee (REC: MS_2020-029a) and following this, online Zoom^©^ semi-structured interviews conducted by trained qualitative researchers were organised with participants at a suitable time for the family and upon gaining the parents” written consent and child’s assent. Parents and children were interviewed separately, allowing for both sets of participants the opportunity to speak freely. Questions were centred around gaining the participants’ thoughts and feelings on the children’s PA and SB within the home and how this was impacted by the COVID-19 restrictions and subsequent easing of restrictions. Questioning followed similar routes using the interview guide (see S2 File), but there was flexibility to react to issues raised by each participant. Question topics included changes in opportunities to be physically active during the lockdown and upon the easing of restrictions and the thought processes behind engaging with these opportunities both within and outside of the home.

### Data analysis

The data analysis process for the first phase of interviews has been previously reported [7] and similar analysis methods were used for the subsequent interviews. The first phase of interviews was analysed before the second and third interviews were conducted. Interviews were transcribed using the automated transcriber on Zoom^©^ and then checked and cleaned. The thematic analysis process outlined by Braun and Clarke [20] was used to analyse the data, utilising a combination of inductive techniques that were driven by the data followed by deductive techniques where factors affecting PA and SB at home were actively searched for. The transcripts were read and re-read (familiarisation) and data of importance was highlighted, including data that suggested novel findings, was repeated across families and that which was related to previous research. Any significant data were then coded using NVivo 12^©^ and reviewed by grouping linked codes together and generating initial and sub-themes in a hierarchical manner. Initial development of themes led the authors to identify concepts of the BPNT, as part of SDT; therefore, the data was mapped in line with this theory to explore the children’s autonomy, competence, and relatedness in the context of their motivation for PA and SB at home across various levels of COVID-19 restrictions. Data obtained from the transcripts authenticated the final theme names that were developed.

Longitudinal qualitative research techniques were explored [21], including Cross-sectional Profiling [22], Case Histories [23], Pattern-Orientated Longitudinal Analysis [24] and Framework Analysis [25]. Of these, the research team decided to use Framework Analysis to analyse the subsequent interviews as it can be used to find patterns across participants, time points and themes [20]. To meet the requirements for the Framework Analysis method, all interviews were analysed by both phase and by family to give two different viewpoints; matrices were created using the Framework Matrix function in NVivo 12^©^ (see S3 File). The restriction timeline was labelled by four categories: lockdown one (March 2020 –June 2020), easing of restrictions (July 2020 –October 2020), lockdown two (November 2020 –March 2021) and new normal (April 2021 –December 2021).

The researchers followed the strategies to support the quality of analysis proposed by Campbell et al. (2021) [26] by firstly engaging in process of reflexivity, followed by discussions between the research group to ensure trustworthiness and credibility. Whilst lengthy engagement with the interview recordings allowed for the researchers to become fully immersed in the topic and gain a deeper understanding of the factors being explored.

## Results

Following analysis of the data, four themes were generated, a through d. The changes in schooling provision theme was further split into three sub themes.

- Changes in Schooling Provision
  - Physical Education (PE) Opportunities
  - Break and Lunchtime Arrangements
  - Changes in Routines of Lesson Structure and The School Day
- Opening and Closing of Clubs and Societies
- Fluctuations in Attitudes and Motivations
- Equipment Availability and Accessibility

Results are discussed on a theme-by-theme basis and as outlined in the methods, in chronological order consisting of lockdown one, easing of restrictions, lockdown two and new normal.

### Changes in schooling provision

Children spend a considerable amount of time in school; a setting that provides many opportunities to be physically active. During the first lockdown these opportunities were removed by home-schooling, which had few, if any, break or lunchtime PA opportunities, no active travel to school or moving between classrooms. Children also encountered mixed experiences of PE provision with some schools providing limited PE opportunities online, some providing theory lessons, whereas some schools did not offer any PE curriculum.

### Physical education opportunities

PE provides an opportunity to promote PA within the school day. During the first lockdown PE was taken away from children, as the closure of schools meant children were being home-schooled. There was a mix of opportunities within PE available for the children to complete in the home environment. Some children were given theory only lessons, whilst others were given videos to watch as a replacement for PE; this was largely unmonitored and not compulsory.

*“I think he had some kind of theoretical stuff to do. But I was never aware of any practical stuff*.*” (Mother, High SES)*

*“Yes*, *they did*. *XXXX had whenever she should have had PE there was an hour’s worth of things in there*, *yoga and some exercises*…*XXX also had a similar thing he had things to do you know that list of different YouTube videos to watch that they work out to*.*” (Father*, *High SES*)

When restrictions were easing and children were returning to school, there were, again, mixed experiences of returning to PE. Schools had restrictions in place, which reduced the PE opportunities available, whilst some schools continued with a theory only approach. One positive change, in most schools, was that changing rooms were out of bounds to prevent the spread of COVID-19, meaning that children wore their PE clothing to school on days with PE. Children preferred this as it was more comfortable than uniforms, they did not have to change in front of their peers, and it gave them more time in PE lessons to be active as there wasn’t a delay from getting changed.

*“No*, *I didn’t [have PE]*. *I would go into class and just do writing about PE*.*” (Boy*, *Aged 14*, *Medium SES*)

*“They’re [PE lessons] much better now like we do football*. *We don’t do rugby because of contact*, *but we are allowed to do football and cricket and stuff like that…No we don’t [wear our uniform to school] just because the changing rooms*, *they don’t want a lot of people inside so we just come in in our kit and we go straight out…it’s a lot more comfortable than school clothes so I do find it much better…it gives you a lot more time like 10/15 minutes more*.*” (Boy*, *Aged 14*, *Medium SES*)

During the second lockdown, schools seemingly provided more at home PE opportunities; increasing children’s physical activity opportunities at home.

*“So mainly just sending like videos and you decided then what you wanted to do like yoga or Joe Wicks or stuff like that*.*” (Girl*, *Aged 13*, *High SES*)

*“We got PE homework in the second lockdown*. *He didn’t particularly like doing it because it was filming*, *he doesn’t like filming himself*, *so he’d do the activities like “keepy uppies” in the garden…but he didn’t really like sending it in…because [he] didn’t want to be on film*.*” (Mother*, *High SES*)

The new normal has provided an opportunity for a re-think of PE in some schools with some continuing to ask children to wear their PE clothing to school on PE days, saving time, infection risk and increasing body-confidence within PE by not having to change in front of peers. Some schools give their children more autonomy to choose their activity and the groups that they participate in, this seemingly has a positive impact on children’s experiences within PE.

*“Um*, *lots of stuff we’ve been doing dodgeball*, *volleyball*, *football*, *netball…we go in our uniform now*.*” (Girl*, *Aged 14*, *High SES*)

*“Well*, *since the virus we’ve had to wear our PE kits into school and that I’m thankful for and I’m glad that we haven’t had to go back to changing in school*. *And the PE lessons have gotten a lot more diverse with having a mixed group*, *girls*, *boys and two different PE things we do*. *But yeah*, *that’s…what I prefer to do rather than what happened in Year 7*. *Before it was based on athletic skill*, *but now it’s more girls*, *boys and mixed but you get to choose which one you prefer to go in*. *They have competitive girls and competitive boys so it’s sort of would you rather be more competitive with people who are the same gender with you*, *or would you rather just be in a sort of more level group of people who are just you know it’s mixed*, *so you have both genders*.*” (Girl*, *Aged 13*, *High SES*)

*“When we came back there was no PE activities it was just writing*. *But now we are able to play football and rugby full contact and everything…we are allowed to use the changing rooms now*.*” (Boy*, *Aged 14*, *Medium SES*)

### Break and lunchtime arrangements

Before the pandemic, break and lunchtimes at school provided an opportunity for unstructured or structured PA for children. This opportunity was removed when children were faced with home-schooling and some children started to spend all day in front of the screen without a suitable break; this minimised their PA within the home.

*“So*, *it [PA] was a lot less than normal…when they’re at school*, *they play rugby at lunch times and all this kind of stuff [but he can’t do that at home]” (Mother*, *High SES*)

During the easing of restrictions and returning to school, there were still many break and lunchtime restrictions in place that the children had to follow. Children reported less time, less PA opportunities and physical structures such as metal barriers and cages that inhibited their movement on the playground.

*“We’d have a corner of the school*, *and we just have to stay there at all times*. *That’s really it…I’d mainly just be sitting down*. *We don’t really have that much room to play sports or anything so… just be talking to people*.*” (Boy*, *Aged 14*, *Medium SES*)

*“Yeah*, *I think that initially when they went back*…*they were in cages when they went out in breaktime and lunch time*.*” (Mother*, *High SES*)

During the second lockdown the poorer weather led to even fewer breaks outside at home for the children. Generally, however, schools had learnt from the first lockdown and provided more of a structured school day for the children at home, giving them opportunities for breaks.

*“They went on Zoom from like half eight till three when school finished*.*” (Mother*, *High SES*)

In the new normal, break and lunchtimes had returned to normal, albeit a little shorter to compensate for the lost lesson time incurred during lockdowns.

*“They do their training during lunch breaks*.*” (Mother*, *Medium SES*)

*“They love it now I think the*, *all the boys and girls in their year group just all play football together all the time now and in lunchtimes and breaktimes and it’s so nice that they’re just completely back to normal now so yeah he’s really enjoying school*, *which is*, *which is good*.*” (Mother*, *High SES*)

### Changes in routines of lesson structure and the school day

Changes that took place from March 2020 and December 2021 in the routine of lesson structure and the school day both increased and decreased time available to participate in PA. During the first lockdown, children reported having more time to be physically active at home as the schoolwork set was flexible and they weren’t too tired during the day. The flexibility allowed them to use outdoor areas, such as the garden, or exploring the local area when the weather was fine.

*“It’s all the schoolwork was on was on Google classroom*. *So it’s all laptop based*. *So they’re on the screens then for three hours a day*.*” (Mother*, *High SES*)

However, upon easing of restrictions, aspects of PA such as movement between classes, break and lunchtimes were reduced. With the children returning to school, they were tired when they got home from a long day of structured lessons, so did not want to be physically active within the home and just wanted to use their time at home to relax, largely on screens.

*“Not really*. *It was you stay in the same class*. *You only get out for break and lunch*, *and it was like you*, *the teachers come to you*, *instead of you go to the teachers normally*.*” (Boy*, *Aged 13*, *High SES*)

During the second lockdown the school day seemed to be less flexible as live lessons became the norm and therefore there was more structure to the school day. This was considered a positive change for many; however, it reduced the flexibility of being able to go for a walk when the weather allowed or when a parent had a break from work.

*“Because of the like more strict day of school and everything [I] didn’t have as much time [for PA]*, *I would say as the first lockdown*.*” (Boy*, *Aged 13*, *Low SES*)

Within the new normal the school day seems to have returned to pre-COVID-19 structure, with extra use of screens for work, potentially increasing sedentary behaviours. Many children reported feeling more tired being back at school full-time, giving them less energy for participating in PA after school. They also noted PA during the school day was restricted due to a tight timetable.

*“It’s more back to normal now*, *although most of the work we do now is on the screen at school*, *so it’s still quite a bit [of screen time]*.*” (Girl*, *Aged 13*, *High SES*)

*“She was less active during lockdown*, *and just probably lost motivation and stuff like that…she’s been far more active since September at school*…*I think it [going back to school] rejuvenated her especially being suddenly being able to do this stuff” (Father*, *High SES*)

*“Sometimes I just feel completely exhausted after school and I just want to have a sit down mostly*.*” (Boy*, *Aged 10*, *High SES*)

### Clubs & societies

Pre-COVID-19, many children attended clubs and societies both in and out of school, which increased their opportunities for PA outside of the home. This opportunity was removed during the first lockdown when all clubs and societies were either forced to stop or moved online. Some clubs continued engaging the children through online challenges whereas others ceased activity. These changes meant children spent more of their leisure time within the home being unable to attend community clubs, potentially increasing both their PA and SB at home.

*“His rugby coach sent him like an exercise thing to do every day*.*” (Mother*, *High SES*)

When the restrictions were easing, clubs had a varying timeline of reopening based on the level of risk. Some clubs were able to safely open their doors to the children fairly quickly after the lifting of restrictions, increasing PA opportunities for some. Other clubs that posed a greater risk of infection could not open until later in the pandemic.

*“Yeah*, *they were quite good with the cricket but cricket’s outside and there’s not an awful lot of contact and whatever contact there is it’s easily wiped with a hand sanitiser for the balls*. *So yeah*, *he did have some sports last year*.*” (Mother*, *Medium SES*)

*“He went back to cycling*, *walking*, *doing everything*, *everywhere*. *The only thing that he missed out on was sport didn’t get back to normal*. *So he did miss out on all his football and his rugby you know…you still weren’t allowed teams to get together in teams or groups*, *so he didn’t have that to go back to*.*” (Mother*, *High SES*)

*“It was a bit strange [going back to clubs] because hadn’t done them for such a long time*, *but it was definitely a good thing to do*, *because I needed the exercise and it felt good to just get out and do something*.*” (Boy*, *Aged 13*, *High SES*)

When the second lockdown was imposed, clubs underwent a similar process to the first lockdown where they either closed fully or moved online, once again restricting children’s activities. The new normal has seen a cautious approach to re-starting clubs, both extra-curricular within schools and community clubs. Some schools were hesitant to start extra-curricular activities due to risk of infection and increased health and safety concerns, whilst others were keen to re-establish these as soon as was possible. Children had mixed views of re-joining their clubs; some were keen to go back and get started, others had enjoyed screentime and other sedentary pursuits during the restrictions and were reluctant to return. Some parents also reported an additional economic impact, whereby the cost of attending clubs had increased which further reduced opportunities for children to be physically active.

*“I mean I’m paying £300 a term for them both to swim once a week*, *which is ridiculous*, *but XXXX’s lessons are £15 for 45 minutes because they can only have half the children in the pool that they could before so everyone’s got to pay double because they’re still going to cover their overhead and everything” (Mother*, *High SES*)

*“The only one*, *he done before the pandemic was water polo and he refused to go back to it*, *which was a shame*, *because I think he did enjoy it when it was when he was doing it…She was happy to go back to all her clubs…she’s keen to do pretty much*, *do everything which is…great yeah*.*” (Father*, *High SES*)

*“Yeah*, *everything’s back to normal*, *the football and the rugby after school all the other sports they’re doing as well yeah*. *I can’t even think of anything that’s not normal about it now so yeah*.*” (Mother*, *High SES*)

*“There haven’t been as many after school activities as they were looking back a couple of years*, *I think the secondary schools have been better at restarting after school activities than the primary [schools]*. *Our primary’s being very cautious*, *and they really aren’t doing any sort of after school clubs*.*” (Mother*, *High SES*)

### Fluctuations in attitudes and motivations

The children’s attitudes and motivations changed throughout the COVID-19 pandemic due to them growing older but also the restrictions being imposed on them. In the first lockdown, many children maintained a positive attitude and motivation towards PA, partly due to the novelty of the lockdown and the good weather that was being experienced, meaning they could be physically active in outdoor spaces at home. Some children did lose motivation for PA and preferred the newfound freedom of being allowed to spend more time on screens.

*“I just love doing sports and it keeps me active all the time*.*” (Boy*, *Aged 12*, *Low SES*)

*“Now I have to encourage her to go out a bit more*.*” (Mother*, *High SES*)

*“I really started doing it [bike riding] more in lockdown because I realized how important it is to maintain physical activity and all that*.*” (Girl*, *Aged 12*, *High SES*)

On the easing of restrictions, those children who previously attended organised physical activity sessions, mostly regained any motivation that was lost and re-joined any activities that were allowed to be resumed. Children who did not previously attend any clubs or societies used the opportunity of more time on screens to persuade parents into letting them continue these elevated levels of screentime. This increased screentime may be due to those children who attend a club or society could be more competent at physical activity, therefore promoting their motivation.

*“I’m definitely a lot more happy because sports will go back to normal now such as school and I’ll be able to go out with no mask and stuff like that and act quite normal*.*” (Boy*, *Aged 14*, *Medium SES*)

*“XXXXX seemed to retreat into himself quite a lot and I think his friends did too*, *which was*, *which was sad*, *and it took us as a pair/as his parents and his friends’ parents quite a while to build their confidence back up again*.*” (Mother*, *High SES*)

*“It made me a bit lazier*. *After coming back from like six hours of school didn’t really do much afterwards so yeah*.*” (Boy*, *Aged 13*, *Low SES*)

The second lockdown was seemingly more challenging as the weather was poor and the days were short, meaning few opportunities to be physically active outside. At this point even some of the more competent children’s motivation to be physically active started to decrease and the lack of relatedness with their peers and autonomy to be physically active had negative implications on their PA levels and attitudes towards being physically active.

*“It made me not want to go out and about…I didn’t really want to do anything—I just wanted just to lie down*.*” (Girl*, *Aged 13*, *High SES*)

*“More in the second [lockdown] locked away in his in his room*, *it was hard to…get them out to*, *‘come on we’ll do something come and walk the dogs with us*,*’ or anything*, *it was hard*, *but we did eventually sort of get him out and about it took a long time*. *It was really*, *really difficult…he just wouldn’t*, *didn’t want to come out*…*It was difficult as well*, *because we couldn’t go very far*, *and it was*, *like there’s nothing around here but we manage*, *we did eventually find some routes that we could walk on that were within the distance and they were nice*, *nice walks*.*” (Mother*, *Low SES*)

As the new normal was imposed, this resulted in a mix of attitudes and motivations towards being physically active, some of this was attributed to COVID-19 but also due to an increasing age of the children. Some children showed a lack of motivation to be physically active and failed to return to PA clubs, preferring screen-based sedentary leisure time within the home. Others were rejuvenated with the newfound freedoms and their levels of PA returned to, or exceeded, that pre-pandemic.

*“Yeah*, *she’s really missed them [clubs] she has she loves a bit of competition*, *so that’s been good*.*” (Mother*, *High SES*)

*“Um I was nervous*, *I expect*, *like everybody” (Girl*, *Aged 13*, *High SES*)

*“I do more like after like being locked down*, *maybe think about like physical health and everything*, *so I just started being more active*.*” (Boy*, *Aged 13*, *Low SES*)

*“Umm*, *I think they’ve appreciated recently that they’ve been good that they’ve had these opportunities and they’ve opened up and they seem keen to take them other than XXXX wanting to give up rugby I’ve not detected the level of reluctance*, *I might have expected*, *I mean we’ve lost [in the rugby team] quite a lot of kids haven’t wanted to come back*.*” (Mother*, *High SES*)

### Equipment availability and accessibility

Notably across the pandemic, participants discussed changes in equipment, with initial equipment bought impacting on PA and SB, including bikes, trampolines, laptops, and TVs.

*“Paddling pool was up; she’d go out on her own*. *And play on that and jump on the trampoline*.*” (Mother*, *High SES*)

*“XXXX had a laptop bought for…because we didn’t have enough equipment to use” (Mother*, *High SES*)

*“New TV we bought when we were in lockdown because they both wanted to watch their own TV programmes and there was a bit… fighting so at the beginning of lockdown we bought a telly*.*” (Mother*, *Low SES*)

*“Well*, *we like to go on a trampoline a lot*. *Like sometimes my cousins came over and like we went on the trampoline and played games and was quite fun*.*” (Boy*, *Aged 13*, *High SES*)

Much of the PA promoting equipment was stored after initial use within the first lockdown.

Some was re-used in the second lockdown, but on the whole once opportunities to be physically active outside of the home returned, the PA equipment bought to meet PA needs within the home was obsolete. In contrast, the sedentary-based equipment was consistently used and not similarly discarded.

*“I was doing some weights ‘cause we bought a few weights to use like at home for like a gym*, *so we did stuff like that*, *watched a couple videos then did that*.*” (Boy*, *Aged 13*, *Low SES*)

*“I think she is on it [laptop] more than she was before the pandemic because a lot more homework is online and then because like before the pandemic she didn’t have a laptop whereas she had to get one during so it’s there it’s accessible for her so she’s it’s just much easier for…to go on then*, *and obviously we’ve got the extra computers that we didn’t have before*.*” (Mother*, *High SES*)

*“We got the trampoline over lockdown which isn’t really being used at the moment*.*” (Father*, *High SES*)

*“[They are] in a cupboard*, *we bought him weights and the bar thing that you do pull ups on the door and that kind of stuff and he just does it all at the gym now he doesn’t bother when he’s at home*.*” (Mother*, *High SES*)

## Discussion

The aim of this study was to track changes in the experiences of children’s PA and SB across changes in COVID-19 pandemic restrictions in the context of the SDT. The results show that changes in schooling provision, clubs and societies opening and closing, attitudes and motivations and the availability and accessibility of PA- and SB-inducing equipment all contributed to the shifting levels of PA and SB throughout the pandemic. The changes within these factors were primary influencers of the children’s PA and SB. This study had a particular focus on PA and SB within the home environment, however, as might be expected, when restrictions were eased, the home environment became less important, as children’s opportunities to be physically active outside of the home increased.

As suggested in the results, the basic human needs according to the SDT, of autonomy, competence, and relatedness, were largely not met during lockdowns and only partly in the easing of restrictions, contributing to a lack of motivation to be physically active, and therefore a decrease in PA behaviours, partly sustained in the new normal. The controlling environment posed by the COVID-19 restrictions left little choice regarding an individual’s opportunities to be physically active. The children therefore experienced a deficit of autonomy in their decision making to be physically active. In research conducted into the first phase of the lockdown [7] competence was found to be a motivating factor, those children who were more competent in PA, were more likely to continue being physically active throughout the lockdown. This pattern continued throughout the remaining phases of the pandemic, with a lack of competence seemingly associated with a lack of motivation to be physically active. There was a clear absence of relatedness during the pandemic with children expressing they missed their friends and when permitted, they returned to PA pursuits to be with their friends once again.

Evidence suggests children are more sedentary when they are indoors [27], and more active when they are outdoors [28], which is concerning as children spend most of their time at home and indoors [29] and even more so when the COVID-19 pandemic restrictions were imposed. Research has found that the physical home environment can provide both barriers and facilitators to PA and SB; whilst the home should be considered a dynamic ecological setting as it is socially impacted [30]. Across the lockdown restrictions changes were made to the physical and social environments within the home space. Children were bought both PA- and SB-inducing equipment in the first lockdown to meet the needs of the new lifestyle. However, during the easing of restrictions and subsequently the new normal, the PA-inducing equipment mostly became redundant and was stored out of sight. This trend was not the same for SB-inducing equipment as this seemed to remain accessible within the home. This is a concerning finding for children’s health as previous studies have found that amongst children and adolescents, having a TV in their bedroom was associated with less PA [31], higher likelihood of being overweight [32] and poor school performance [33]. In pre-school children it has been found that the availability of sporting equipment at home did not differ by child weight status [34]. However, a study with adults showed that participants with a higher BMI reported less PA equipment in the home, and that more moderate-vigorous PA was associated with the presence of more PA equipment in the home [35]. A systematic review exploring attributes in the physical environment in relation to children’s PA found mixed results, with four out of six studies showing no associations between home equipment and observed, self-reported and objectively measured PA [36–40]. However, in one study, self-reported PA was positively and significantly associated with the number of pieces of exercise equipment in the home amongst adolescent girls and boys [41].

Schools provide numerous opportunities for children to be physically active and parents alluded to this in the interviews; where changes in schooling provision, including PE opportunities, break and lunchtime arrangements and changes in the routines of lesson structure and the school day, all contributed to the fluctuations in PA and SB during the pandemic. With few to no opportunities through school, PA within the home environment increased but this did not compensate for the overall decrease in PA during the restrictions. PE is important for children to provide opportunities to participate in MVPA, with some studies reporting that 34% of PE lessons are spent in MVPA [42], contributing to the children’s achievement of the PA recommendations [43]. PE lessons are also important for students to acquire knowledge, skills, and motivation to be active outside school and later in life. During the first lockdown children were either not given PE for home-schooling or given limited PE including videos to watch, such as the “Joe Wicks” sessions. Upon the easing of restrictions, children reported either not taking part in physical PE lessons, but PE theory lessons took precedence. If children did have practical PE lessons, no contact sport was allowed, some schools required face masks to be worn, and children wore their PE kit to school. Wearing PE kit to school was a major discussion point during the interviews as children much preferred to do this, as it gave them more time in PE lessons and removed the barrier of embarrassment of changing in front of their peers. This was consistent with previous research which identified PE changing rooms as a problematic space for pupils [44]. This space can be a hub for bullying, if pupils are perceived as ‘different’ [45], and this is further acknowledged by care-experienced children who may have been subject to neglect or abuse [46].

Some schools improved on their PE provision during the second lockdown providing more structured sessions, but some parents still prioritised academic subjects and did not want to create an additional challenge with their child in encouraging them to take part in the PE provided by the school, whilst at home. The new normal has seen varying school rules for PE; on the whole, PE has returned to pre-COVID-19 standards, if not with some changes either due to lessons learnt over COVID-19 or the new health and wellbeing curriculum which has been partly implemented in some schools already but being rolled out nationwide across Wales in September 2023 [47]. Some schools have changed the way they conduct PE lessons so that the children have more autonomy in their PE, choosing between competitive groups, mixed gender groups and PA opportunities. Consistent with previous research, giving children autonomy in their learning, has a positive influence on the learning process [48]. Some schools continue to ask their pupils to wear their PE kit to school, which is a positive change for many, whilst others have returned to changing at school, which is not so highly received. Contact sports are permitted, and the use of face masks has been removed. This return to normal for school PA opportunities has further decreased PA within the home due to an increase in time spent away from the home environment.

Schools provide opportunities through active transport, unstructured play, and extra-curricular clubs. These opportunities were all taken away from the children in the first lockdown when schools were forced to close, leaving children with a lack of autonomy. Upon easing of restrictions, children’s PA opportunities at school were limited with children reporting break and lunchtimes being shorter and spent in small areas, described by some as cages, presumably to aid with social distancing. MVPA and very high PA is higher at break and lunchtime for boys than for girls [49] and the PA accumulated during breaktimes may contribute between 5% and 40% towards the daily PA recommendations [49]. During both lockdowns there was no scheduled time for break or lunch activities and during the easing of restrictions children reported that their PA decreased during break and lunchtime due to limited time and space. Positively, during the new normal phase, break and lunchtime seems to have returned to pre-COVID-19 structure and this has once again become an opportune time for children to accumulate some of their daily PA.

The final changes that were made at a school level which had an impact on the children’s PA and SB were the changes in lesson structure and daily routine. During the first lockdown, parents were given the task of trying to keep their child in a routine, as the schoolwork was set for the children to complete throughout the day, but not at specific times. This absence of routine lead to an increase in screen-time and therefore SB at home [50]. However, this flexibility was removed during the second lockdown when many schools moved to provide live virtual lessons. Some parents commented that this more structured day kept the children in a better routine, whilst others did not find this helpful, as they preferred the flexibility of getting out for a walk when the sun was shining, or when they were having a break from meetings whilst working from home. During the easing of restrictions, the structure of the school day changed to minimise the spread of the virus, children were required to stay in one classroom, and it was the teachers who moved between classes for lessons, rather than the children. Moving between classes is an unstructured form of PA for the children, and associations have been found between larger school sites and increased PA [51]. The large sites mean accumulating PA during the school day just from moving between classrooms, but this was removed during the easing of restrictions. The new normal has meant a return to pre-COVID days where children are once again moving around the school between lessons, accruing PA as they go.

According to the Sport Wales School Sport Survey, pre-COVID-19, 65.1% of children aged 7 to 16 years attended a community sport club at least once per week, whilst 49.9% attended at least one extra-curricular sports club per week [52]. Children spoke about the social aspects of going to these clubs and the relatedness that they feel to their friends who also attend the clubs. Clubs and societies were initially forced to close, with some providing virtual opportunities. There were mixed returns during the easing of restrictions, some clubs opened with ease and others which struggled do so with rule changes. The second lockdown forced the clubs to close again with children losing opportunities for PA once again. These fluctuations in opening and closing of clubs have led to changes within the new normal, including financial impacts. Previous research found that ‘financial situation’ as a barrier to PA reduced during the pandemic [53] but had previously been reported as a significant barrier to children’s participation in PA [54], particularly to single parents [55]. Some parents, including both single parents and parents from nuclear families, said that during the new normal, prices had increased to a level where they could now, unfortunately, only provide limited opportunities for their children, this was particularly prominent with swimming clubs. This is a concern, as more than two-thirds of families in this study were of a high SES and they were struggling to afford swimming, so lower SES families may be more impacted by this and it is known that children learning to swim is of paramount importance for fitness [56], health and safety [57]. Some children, generally the more physically competent ones, could not wait to return to their clubs to feel a sense of relatedness. In contrast, others were not motivated to return and found alternative leisure pursuits during the lockdowns, mainly involving SB within the home environment.

Not being motivated to return to active leisure pursuits when the opportunity arose was observed in the fluctuations in attitudes and motivations to be physically active throughout the pandemic. Parents commented on the changes in their children’s attitudes throughout the lockdown; some attributed this to their age, which is consistent with previous findings that children’s PA reduces with age [28]. Parents discussed that the changes in their children’s motivation and attitudes may have been down to increasing age, but also due to the COVID-19 restrictions. During the first lockdown, motivation and attitudes towards PA were generally positive as it was a novel time for the children and parents alike. Outdoor PA pursuits such as walking and cycling were popular as the weather was dry and warm, which has been previously positively associated with PA [58]. After the first lockdown, the easing of restrictions occurred during the summer months, again with limited rainfall, this meant that the children’s PA seemingly increased from the lockdown as there was more freedom in a less controlling environment, leading to more autonomy; this is supported by the finding that children’s PA levels significantly increased upon return to school [59]. The second lockdown posed new PA considerations for families, as the weather was poor, and the daylight hours were shorter further reducing opportunities for PA outdoors. This had a negative impact on children’s motivation to be physically active. The children reported being “tired” and “bored” of lockdown and in some cases, this had a negative impact on wellbeing and mental health. During the new normal phase there were varying attitudes towards returning to pre-COVID-19 PA levels. Some children became even more physically active due to missing out during the restrictions or having a greater understanding of the importance of physical health; these were generally the more physically competent children. Other children gained motivation due to social aspects and relatedness; they wanted to be physically active with their friends again [60]. Some children continued with more sedentary pursuits, often involving screentime, potentially due to a lack of motivation stemming from the restrictions. This was also documented in a study measuring PA pre, during and post-lockdown restrictions, where there was a decrease in PA and an increase in SB [12].

### Strengths & limitations & future research

When considering the outcomes from this research, several strengths and limitations should be acknowledged. To the authors’ knowledge, this is the first longitudinal qualitative analysis of children’s PA and SB during the COVID-19 pandemic, where both parents and children shared their experiences. The longitudinal nature of the study allowed the researcher to follow up on specific details of prior interviews to delve deeper into the participants comments’ and generate case studies of families’ experiences. This may also have built additional levels of trust between the participants and the researcher, reassuring them to share more information. It allowed for a timeline of changes in PA and SB to be created for each family to gain a more thorough understanding of the perceived changes in children’s PA and SB across various timepoints of the pandemic, allowing comparisons to be made between different lockdowns and easing of restrictions.

However, this study was not without its limitations, including the attrition of participants. As outlined in the methods, there were 20 families interviewed in the first phase, 14 in the second and nine in the third, which included two sibling dyads. This is a 55% drop out rate from the first to the last phase of interviews. Despite this dropout, both boys and girls, mothers, and fathers and at least one family from high, middle, and low SES were interviewed in the final phase. Reported rates of attrition within longitudinal studies range from 30% to 70% [61], therefore, this study falls in the average range of drop out. Due to the volunteer uptake, there was an overrepresentation of high-SES families which is common in research [62, 63]; two-thirds of the families were from high-SES as calculated by the WIMD. However, comparisons can be made between families of similar SES, living in similar environmental conditions. An additional limitation includes the difference in researcher between the first interview and second and third interviews where there was consistency. Finally, the study did not report objectively measured PA or SB and relied on self-report to understand the changes in PA and SB during the COVID-19 pandemic.

Therefore, future research should seek to provide a mixed methods approach to PA and SB changes at home to incorporate the use of accelerometers to objectively measure these behaviours. Low SES families should be prioritised, and comparisons could be made in PA and SB at home between families with varying levels of affluence. Although beyond the scope of the present study, future research should seek to see if the changes have remained consistent post-pandemic and whether, as these children age, there is a long-term impact of the COVID-19 pandemic restrictions on PA and SB as they move into adulthood.

## Conclusion

Use of the SDT allowed us to consider the factors involved in autonomy, competence and relatedness that influenced the motivation behind the children’s increases and decreases in PA and SB. It was clear that imposing a controlling environment on the children largely decreased their PA opportunities and subsequently their motivation to be physically active. Furthermore, this decreased their levels of autonomy and relatedness as they could not participate in PA with others, this lack of a social element further decreased PA. The results of this study provide an insight into the changes in PA and SB throughout the varying levels of restrictions in the COVID-19 pandemic. Changes in schooling provision, clubs and societies opening and closing, attitudes and motivations and the availability and accessibility of PA- and SB-inducing equipment all contributed to the fluctuating levels of PA and SB throughout the pandemic.

In terms of applied implications should children have to spend a considerable amount of time in their home environment again in the future, measures should be taken to provide alternative opportunities to be physically active, both indoors and outdoors. The findings can further inform the need to reverse these decreases in PA through ensuring that children’s voices are heard, particularly about PE lessons. Although PA has increased following the lockdowns, it is imperative that children are given a range of opportunities to be physically active to reverse any negative impacts of the COVID-19 restrictions.

## Supporting information

S1 File The standards for reporting qualitative research checklist. (DOCX)

S2 File The interview schedules. (DOCX)

S3 File The minimal dataset in the form of a Framework Matrix. (XLSX)
