# Supplementary material for: Qualitative changes in children’s physical activity and sedentary behaviours throughout the COVID-19 pandemic: The HomeSPACE project
Source: PLoS One. 2023 Jan 20;18(1):e0280653. doi: 10.1371/journal.pone.0280653 (PMC9858462; doi:10.1371/journal.pone.0280653)
Supplement: S2 File — (DOCX) [file pone.0280653.s002.docx]

HomeSPACE interview questions – Phase 1 of Interviews

Four pre-identified themes will be used to guide the interviews; **(1)** perceptions of the children’s PA and sedentary behaviour during the pandemic compared to before, **(2)** opportunities and challenges in keep their children active during the pandemic, **(3)** factors influencing PA and sedentary behaviour during the pandemic, **(4)** changes in the physical and social environment in response to the pandemic and perceptions of how they might be influencing PA and sedentary behaviour at home.

# Children’s perceptions of physical activity and sedentary behaviour and their influences at home

# Usual Activities - Card Sort Activity (Children only)

- I'm going to ask you to talk about what you do on a usual weekday. Is that okay?
- There's a pack of cards in front of you - take a look at the cards and choose the 3 activities that you do most on a typical school day outside of school hours – so morning, afternoon and evening. Then we'll talk about them. *Prompt: Start in the morning, afternoon, evening. Think about yesterday.*
- Which activity do you do most? Where do you do this? Do you do it with others or by yourself? *Do for all activities.*
- *Ask how and why this is different from before the COVID-19 restrictions, make sure to cover all questions (I.e., which activity do you do most, where do you do it, do you do it with other or by yourself)*
- And what about a usual weekend day? Take the cards and select 3 cards based on a usual weekend day. *Prompt: Start in the morning, through the day, and evening? Think about last Saturday.*Which activity do you do most? Where do you do this? Do you do with others or by yourself? *Do for all activities.* Which do you like best?
- *Ask how and why this is different from before the COVID-19 restrictions, make sure to cover all questions (I.e., which activity do you do most, where do you do it, do you do it with other or by yourself)*

# Factors influencing PA and sedentary behaviour ( For child)

- What helps you to be more active at the moment?
- What makes you less active/ lazy (sitting playing video games, watching TV)? (Maybe separate for weekday and weekend days)

# Parents perception of their children’s physical activity and sedentary behaviour

- If you were to describe your child’s level of physical activity at home at the home as low, medium or high, which one would you pick?
- *Remind them that PA is “anything that gets the body moving-it can be in the form of structured exercise or it can be free play, running around the garden”*
- Can you tell me more about why you picked x?
- Why do you think that?
- Compared to before the pandemic, are they less or more active? Why?
- What about screen time/screen viewing? *Tell them that screen viewing includes time spent computers, laptops, games consoles, ipads, mobile phones, not just TV.*
- If you were to describe your child’s level of screen viewing on weekdays as low, medium or high, which one would you pick?
- Can you tell me more about why you picked ‘X’? Why? (do you think that)
- What about weekend days? Would you describe their screen viewing on weekend days as low, medium or high?
  - Can you tell me more about why you picked ‘X? Why? (do you think that)
- Compared to before the COVID-19 restrictions, do they spend more or less time screen viewing? Why?

# Factors influencing PA and sedentary behaviour (For parent)

- What are the main things that decide how active your child is at home? Think about individual, social and physical factors? (need to think of a way to word this so that parents understand)
- What affects the amount of time your children spend watching TV or using computer games when at home? Think about individual, social and physical factors?
- Any changes because of the covid-19 restrictions?

# Opportunities and challenges in keeping your children active during the restrictions (Family)

- Is increasing your children’s physical activity at home important to you?
- Currently (with Covid-19 restrictions), how achievable/possible is it? Why? in what way?
- Is managing your children’s screen viewing at home important to you?
- Currently (with Covid-19 restrictions), how achievable/possible is it? Why? in what way?

# Home tour (Family)

Now I'd like to talk with you about the different places in your house and yard that you use for different activities. I’d like to do this as a moving tour. What that means is I’ll ask ________ to think of places in your house and yard where he/she spends the most time. Then we’ll go to those places and you can tell me all about the place and how you use that place. It’s completely up to you where the tour goes. You are in charge. And we can come back here at any time. Does that sound okay?

_________, think of the three places in the house and yard where you spend the most time when you are awake. What are they? What about we start in the ___________ room?

# For Each Room... (Family)

- Tell me about this area/room?
- What types of activities happen in here?
- How much time do you spend in here?
- Who are you usually with?
- What equipment do you have in here (*prompt: media, play equipment, seating*)? Is it easy to get to the equipment you have?
- Has anything changed in this room in response to the COVID-19 restrictions? Why? Talk about how it’s influencing your child’s PA/screen-viewing?
- Are there any other places that have changed in response to the COVID-19 restrictions that you would like to show me? Why? Talk about how it’s influencing your child’s PA/screen-viewing?

HomeSPACE interview questions – Phase 2 of Interviews

# Introduction

- Researcher to introduce self and thank participants for their time
- Ask to speak to all participants for a few minutes to outline the discussions
- Last time they were interviewed, the main focus was on the first lockdown in March 2020
- Today we want to split the discussion into two main timepoints the first being last summer when restrictions started to lift through the summer holidays to going back to school in September. And the second timepoint is when the restrictions came back in around October time through to March 2021.
- Remind participants that researcher will remind them which timepoint we are discussing
- Reminder that if there is anything they don’t want to discuss or answer anything they don’t have to and we will be recording the session, haven’t started yet will let them know when start. And if they don’t understand something or want reminding of a question to just ask. No right or wrong answers, we just want to explore how your physical activity and sedentary behaviour (sitting down) changed as the restrictions changed.
- Start recording.
- Ask for a reminder of children’s ages, who they live with including any pets.
- Ask when the children went back to school. Before summer holidays? After fire break? Before Easter this year?
- Has anyone in the house had to isolate?
- Ask to speak to participants individually.

# Child – Lifting of Restriction

- Refer back to if the child went back to school before the summer holidays
- How did you feel knowing that the restrictions were lifting? And going back to school? Why did you feel like that?
- How did you find the summer holidays? What did you do?
- Did you go to any clubs over the summer? Holiday?
- Did you go back to after school clubs when you were allowed to?
- How did you feel about that? Did you have any motivation to go back to clubs?
- How did your friends feel and what did they do when the restrictions were lifts? Did they go back to clubs? Did you see them outside?
- How do you think your physical activity at home changed?
  - Did you carry on going on walks etc?
- How do you think your screen time at home changed?
  - Did you carry on gaming when you could?
  - Did your friends?
- What was PE like when you went back to school?
- Did you go back to any community centres/leisure centres?
- Social/environmental factors to be physically active or spend time on a screen?
- Equipment at home?
- Did you play games at home as a younger child? Did you have equipment to do this? Did you go back to any of these games during lockdown to prevent boredom?

# Child – Lockdown Restrictions

- October/November we went back into lockdown restrictions
  - How much did you know about the restrictions?
  - How did you feel?
  - How did you feel not going to school again?
- We sort of knew what was coming, were you looking forward to anything?
- What were you not looking forward to?
- Did you learn anything in the first lockdown that you used to help you in the second lockdown?
- Thinking about your routine and time at home, was anything the same as the first lockdown?
- Was anything different?
- First lockdown – PA was *researcher to read from first summary*, this changed when the restrictions were lifted, how would you say it changed again in the second lockdown? Higher or lower?
- SAME AS ABOVE FOR SED BEH/SCREENTIME. Higher/lower?
- Talked about things that made you want to be PA during lockdown did this change in the second lockdown? Did anything make you be physically active?
- Talked about things that made you want to spend time on a screen during the lockdown, did this change in the second lockdown? Anything make you want to spend more time on a screen?
- Christmas holidays
  - How did you feel?
  - How different to previous Christmas?
  - Physical activity during Christmas? Sedentary Behaviour during Christmas?

# Parent - Lifting

- How did you feel with the lifting of the restrictions?
- How do you think your child felt?
- Refer to when child went back to school.
- How were the summer holidays?
  - Did you get away at all?
  - Did the children go to any clubs?
  - Did they manage to see friends a bit more often?
- Did you notice that they wanted to go out more than before the restrictions? How did their attitudes/motivation change?
- During lockdown PA was **refer to previous interview** how did that change when the restrictions lifted?
- During lockdown screen time was **refer to previous interview** how did that change when the restrictions lifted?
- We discussed factors that made them active during the lockdown, but what about when restrictions were lifted?
  - Were there any factors that made them more active both outside of home and in the home?
  - Were there any factors that made them spend more time on the screen?
- Did you keep any sort of routine?
- Did you change anything based on your experiences during the restrictions?
- Did you face any challenges around PA/Screen time when the restrictions were lifting?

# Parent – Lockdown

- Oct/Nov we sort of knew what was coming next and we went back into lockdown.
- How did you feel?
- How did the children feel?
- What happened with home schooling?
- First lockdown – PA was *researcher to read from first summary*, this changed when the restrictions were lifted, how would you say it changed again in the second lockdown? Higher or lower?
- SAME AS ABOVE FOR SED BEH/SCREENTIME. Higher/lower?
- Talked about things that made you want to be PA during lockdown did this change in the second lockdown? Did anything make you be physically active? Weather not good!
- Talked about things that made you want to spend time on a screen during the lockdown, did this change in the second lockdown? Anything make you want to spend more time on a screen?
- Challenges around PA during this second lockdown?
- Challenges around screen time during the second lockdown?
- Routine?
- Did you stick to the restrictions as closely as the first lockdown?

# Conclusion

- Last questions:
- How do you feel about the future?
- Looking forward to the future are there any changes that you would make based on the last 18 months
- Researcher to thank participants for their time
- Will send over consent form to allow for the interview to be used in our analysis
- Be in touch again in September/October to hopefully discuss the “new normal”

HomeSPACE interview questions – Phase 3 of Interviews

# Introduction

- Researcher to introduce self and thank participants for their time
- Ask to speak to all participants for a few minutes to outline the discussions
- First time they were interviewed, the focus was on the first lockdown in March 2020 = July 2020
- Interviewed again during summer 2021 to discuss the changes moving in and out of lockdown
- Today we want to focus on what we call the “new normal” during the summer and into September through to now.
- Reminder that if there is anything they don’t want to discuss or answer anything they don’t have to and we will be recording the session, haven’t started yet will let them know when start. And if they don’t understand something or want reminding of a question to just ask. No right or wrong answers, we just want to explore how your physical activity and sedentary behaviour (sitting down) changed as the restrictions changed.
- Start recording.

# Children

- Ask for age and reminder of who live with
- Spoke at start of July and were looking forward to the summer and going out with friends on bikes etc with almost all the restrictions being lifted. Can you remember what summer was like?
- School – September - what was it like going back to school? How did you feel? What was PE like? How did you get to school? Break/lunchtimes? After school clubs? Isolate from school?
- What do you do at home when you get home from school? Is this different to before the pandemic? Or during the pandemic?
- Weekends since September, what have they looked like?
- Did you start doing anything during lockdown that you have carried on doing now?
- Screen time? Gaming?
- Where do you do most of your physical activity?
- Play? Parks? Community Centres? Leisure Centres?
- Equipment?
- What now motivates you to be physically active?
- What makes you not want to do physical activity?
- Weather
- Before the pandemic what was important to you? Still important now?
- What were your views on physical activity before the pandemic? Are they still the same?
- What were your views on screentime before the pandemic? Are they still the same?

# Parents

- Summer
- How do you think your child felt going back to school in September?
- What differences did they talk about? PE? Afterschool clubs? Activities when got home?
- Community Clubs?
- How would you compare their PA now compared to before Covid? Why do you think this is?
- How would you compare their screentime now to before Covid? Why do you think this is?
- What are the main differences in their PA/SB now compared to both during and before Covid?
- Last time we discussed factors that made them active. What would you say these are now?
- We also discussed factors that made them less active. What would you say these are now?
- At home and outside the home?
- Do you do anything now that you wouldn’t have done if it weren’t for Covid?
- We also discussed any challenges around PA/Screen time – what do these look like now?
- Main differences in your child’s activities at home?
- Weather?
- Before the pandemic what was important to you? Still important now?
  - Physical activity important?
  - Screentime important?

# Concluding Comments

- Last questions:
  - How do you feel about the future?
  - Looking forward to the future are there any changes that you would make based on your experiences with the pandemic?

Researcher to thank participants for their time
